# ICD-10 coding algorithms for defining comorbidities of acute myocardial infarction

**DOI:** 10.1186/1472-6963-6-161

**Published:** 2006-12-15

**Authors:** Lawrence So, Dewey Evans, Hude Quan

**Affiliations:** 1 Department of Community Health Sciences and Centre for Health and Policy Studies, University of Calgary, Calgary, Alberta, Canada; 2 Department of Economics, University of Calgary, Calgary, Alberta, Canada; 3 British Columbia Cardiac Registry and Evaluation Services, Provincial Health Services Authority, Vancouver, British Columbia, Canada

## Abstract

**Background:**

With the introduction of ICD-10 throughout Canada, it is important to ensure that Acute Myocardial Infarction (AMI) comorbidities employed in risk adjustment methods remain valid and robust. Therefore, we developed ICD-10 coding algorithms for nine AMI comorbidities, examined the validity of the ICD-10 and ICD-9 coding algorithms in detection of these comorbidities, and assessed their performance in predicting mortality. The nine comorbidities that we examined were shock, diabetes with complications, congestive heart failure, cancer, cerebrovascular disease, pulmonary edema, acute renal failure, chronic renal failure, and cardiac dysrhythmias.

**Methods:**

Coders generated a comprehensive list of ICD-10 codes corresponding to each AMI comorbidity. Physicians independently reviewed and determined the clinical relevance of each item on the list. To ensure that the newly developed ICD-10 coding algorithms were valid in recording comorbidities, medical charts were reviewed. After assessing ICD-10 algorithms' validity, both ICD-10 and ICD-9 algorithms were applied to a Canadian provincial hospital discharge database to predict in-hospital, 30-day, and 1-year mortality.

**Results:**

Compared to chart review data as a 'criterion standard', ICD-9 and ICD-10 data had similar sensitivities (ranging from 7.1 – 100%), and specificities (above 93.6%) for each of the nine AMI comorbidities studied. The frequencies for the comorbidities were similar between ICD-9 and ICD-10 coding algorithms for 49,861 AMI patients in a Canadian province during 1994 – 2004. The C-statistics for predicting 30-day and 1 year mortality were the same for ICD-9 (0.82) and for ICD-10 data (0.81).

**Conclusion:**

The ICD-10 coding algorithms developed in this study to define AMI comorbidities performed similarly as past ICD-9 coding algorithms in detecting conditions and risk-adjustment in our sample. However, the ICD-10 coding algorithms should be further validated in external databases.

## Background

Acute myocardial infarction (AMI) outcomes are studied frequently in health service research with hospital discharge administrative data [1-8]. Risk adjustment is an important tool used in health service research to account for differences in AMI patient's characteristics. To develop such a tool for AMI patients, Tu et al. [3] initially selected 43 comorbidities that were used as potential risk factors for AMI in United States hospital report cards. The nine comorbidities were chosen from the 43 comorbidities based on their clinical plausibility and statistical significance. Along with adjustments for sex and age, these 9 comorbidities were found to substantially predict AMI mortality in Ontario provincial database, and were validated in 2 external databases from Manitoba, Canada and California, the United States. Subsequently, many studies have assessed its validity and employed the AMI prediction method [4,5,9-21]. Grunau et al. compared Tu et al's disease specific prediction rule to many other methods [20]. These authors found that Tu's method performed better than other general methods that summarized comorbidities as a score, or included comorbidities that are less relevant for AMI outcomes. Hence, Canada has accepted Tu's risk adjustment method to generate public reports of AMI outcomes [21].

With the introduction of tenth revision of the International Classification of Disease (ICD-10) coding system by the World Health Organization (WHO) [22] in 1992, this risk adjustment tool may no longer be valid. ICD-10 is significantly different from ICD-9 coding system, differing in the richness of clinical information and use of alphanumeric codes relative to numeric codes in ICD-9. Hence, previously validated [23] ICD-9 coding algorithms used for the nine comorbidities cannot be applied to the new ICD-10 data. Without valid ICD-10 coding algorithms, administrative data can not be used to measure and control for patients' comorbidities when studying their clinical outcomes. Further, future policy and quality decisions using ICD-10 data require the development and validation of ICD-10 coding algorithms for these comorbidities.

The objective of this study was to develop ICD-10 coding algorithms for the nine AMI comorbidities. The validity of these newly developed ICD-10 algorithms was determined by reviewing medical charts in recording AMI comorbidities. We then applied the ICD-10 and past ICD-9 coding algorithms to administrative data from a large Canadian province. Comparisons between the two coding algorithms were made by comparing their performance in predicting in-hospital, 30-day, and 1-year AMI mortality.

## Methods

### Development of ICD-10 Coding Algorithm

A three step process was employed to develop the ICD-10 coding algorithms for the nine AMI comorbidities, which include shock, diabetes with complications, congestive heart failure, cancer, cerebrovascular disease, pulmonary edema, acute renal failure, chronic renal failure, and cardiac dysrhythmias [3]. In the first step, the ICD-10 Canadian computerized code finder was used to generate a list of all relevant ICD-10 codes, matching to clinical terms of the comorbidities. Two coders trained in both ICD-9 and ICD-10 coding systems independently generated separate lists, which were then compared to form a single list of ICD-10 codes. A physician was consulted for the disagreements between the two coders on the codes.

In the second step, ICD-9-CM coding descriptions from the ICD-9-CM manual were used to generate a list of matching ICD-10 codes. Again, each coder independently generated a list of ICD-10 codes, which then were compared to form a single list. This second list was merged with the list from step one to form a comprehensive ICD-10 code list. In the third step, four physicians independently reviewed this list, and then met to determine whether each coding algorithm met the clinical meaning based on their experiences. The four physicians are specialists in neurology, general internal medicine, and psychiatry. For the disagreements about the codes on the list, an additional physician was consulted to finalize the list of coding algorithms for the nine comorbidities.

### Alberta Chart Review data

A simple random sample from four teaching hospitals was used to select 4,008 patients' charts to be reviewed. These records were patients who were discharged between July and December 2003 in the province of Alberta, Canada. In this study period, hospital discharge data were coded in ICD-10. Two reviewers with coding and medical training reviewed the charts and determined the presence of the nine comorbidities. Moreover, the charts were re-coded by regular health record coding staff in ICD-9 to create a 'real world ICD-9 dataset'. Therefore, three databases were generated for the same charts, including the originally coded ICD-10 data, chart review data and re-coded ICD-9 data. Of these 4,008 records, there were 193 patients with AMI, based on their chart information. We included these 193 patients for assessment of validity of ICD-9 and ICD-10 data in recording the nine comorbidities.

### ICD-9 and ICD-10 Administrative Data

We used hospital discharge abstract data from 1994 to 2004 fiscal year from the province of British Columbia, Canada. This administrative data was coded in ICD-9 between 1994 and 2000 fiscal year, and then was coded in ICD-10 between 2001 and 2004 fiscal year. To define AMI patients, we abstracted patients with the most responsible diagnosis field coded as 410.x in the ICD-9 data and I21.x and/or I22.x in ICD-10 data. In addition, we kept all patients with coronary artery disease (those with the most responsible diagnosis field coded as 414.0 in ICD-9 and I25.1 in ICD-10), AMI (the secondary diagnosis fields coded as 410.x in the ICD-9 data and I21.x and/or I22.x in ICD-10 data) and underwent coronary angioplasty or bypass graft surgery (48.1, 48.02, 48.03 of Canadian Classification of Procedure (CCP) [24] for ICD-9 data and 1IJ76 of Canadian Classification of Intervention (CCI) [25] for ICD-10), but excluded patients who underwent heart valvuloplasty surgeries (47.1 and 47.2 of CCP for ICD-9 and 1HV80, 1HU80, 1HT80, 1HS80, 1HS90, 1HT90, 1HU90, 1HV90 of CCI for ICD-10 data). Furthermore, we excluded AMI patients with the exclusion criteria: 1. not a British Columbia resident, 2. age less than 20 years, and 3. length of hospital stay less than or equal to one day. Patients who are discharged on the same day of the admission or the following day were excluded to remove emergency room deaths and to improve the accuracy of AMI diagnosis. After excluding patients meeting these criteria, patients were sorted by their earliest AMI admission. Admissions following the earliest admission or admissions in 1994 were excluded. Finally, these AMI patients were linked with British Columbia vital statistics to determine deaths after discharge.

### Statistical Analysis

In order to validate the ICD-9 and ICD-10 coding algorithm, we compared ICD-9 and ICD-10 data with chart review data in defining comorbidities. Chart review data was used as a 'criterion standard'. Sensitivity, specificity, positive predictive value (PPV) and negative predictive value (NPV) statistics were calculated for comorbidities in ICD-9 and ICD-10 data.

After validating the ICD-9 and ICD-10 coding algorithms, we applied them to British Columbia's administrative data and produced descriptive statistics for the study population. Logistic regressions were used to predict AMI mortality with ICD-9 and ICD-10 coded comorbidities. An alternative modeling approach is to use a Cox regression to account for the risk of mortality over time. However, logistic regression was used to predict mortality in the fixed period. This approach has been commonly used in previous studies. Therefore, comparability of our study to previous studies could be increased by using the logistic regression approach. Using the above nine comorbidities and the study populations' age and sex, we predicted in-hospital, 30-day and 1-year AMI mortality. Each OR should be interpreted as controlling for all nine comorbidities, age and sex. No model building strategy was employed because this 11 variable prediction rule had been validated by Tu et al's study and referenced subsequently by studies using this prediction rule [4,5,9-21].

The regressions between ICD-9 and ICD-10 coded comorbidities were evaluated by comparing their C-statistics, Brier Scores, and R-squares. A C-statistic evaluates each model's ability to discriminate deceased from alive patients by comparing the observed to predicted values. A C-statistic equal to 0.5 indicates random prediction, while a value of one indicates perfect prediction [26]. If the C-statistics generated from ICD-9 regressions are similar to the C-statistics from ICD-10 regressions, then we could infer that ICD-10 coded comorbidities perform comparably to ICD-9 coded comorbidities. Another measure we used to assess the models' predicted probabilities was the Brier score. The Brier score measures the mean squared difference between expected probability of dying and its actual occurrence [27]. Low Brier scores indicate that there is a small difference, which shows that the model predicted well. The R-square summary statistic is used to assess how well the model fits with the data. In particular, R-square measures the proportion of the total variation in mortality explained by the logistic regression model.

## Results

### Agreement between Administrative Data and Chart Review Data

Table 1 shows ICD-9 and 10 coding algorithms for defining the nine comorbidities. The comparison of ICD-9 and our ICD-10 coding algorithms and medical charts is shown in Table 2. Frequencies for six AMI comorbidities were similar among ICD-9, ICD-10 and chart review data. Three comorbidities, i.e. cerebrovascular disease, pulmonary edema and cardia dysrhythmias had similar frequencies between ICD-9 and ICD-10 data, but lower frequencies compared with chart review data. Each comorbidity recorded in ICD-9 and ICD-10 datasets was very similar in sensitivity, specificity, PPV and NPV (see Table 3). However, the validity varied across comorbidities in both datasets. Sensitivities ranged from 7.1–100% and PPVs ranged from 33.3–100% in both ICD-9 and ICD-10 data, respectively. Specificity for all comorbidities was higher than 93% in both datasets. NPV was low for cardiac dysrhythmias (56.1% in ICD-9 and 55.6% in ICD-10 data) and higher than 86% for the remaining eight comorbidities. The width of the 95% confidence intervals for the nine comorbidities shows the low prevalence for most of the comorbidities in the chart review data except for cardiac dysrhythmias and congestive heart failure.

### Frequency and Mortality in Administrative Data

Given the similarity to chart review data for the majority of ICD-9 and ICD-10 coded comorbidities, we applied the coding algorithms to hospital discharge abstract dataset. There were 29,610 AMI cases in ICD-9 dataset (1995–2000 fiscal years) and 20,251 in ICD-10 dataset (2001–2004 fiscal years). The frequencies for age, gender and comorbidities were similar between the two datasets (see Table 4). Patients were predominantly male (approximately 66%) and older than 50 years old (approximately 90%). Congestive heart failure and cardiac dysrhythmias were the two most prevalent comorbidities, while pulmonary edema was the least prevalent comorbidity. The absolute difference in the frequencies between ICD-9 and ICD-10 coded comorbidities was less than 2%.

The in-hospital, 30-day, and 1-year mortality rates for the AMI patients were 8.5%, 9.9%, and 16.5% in ICD-9 data and 6.8%, 9.0% and 16.4% in ICD-10 data, respectively. Comorbidity specific mortality rates between the two datasets revealed a similarity (see Table 5). Shock and acute renal failure had high mortality. The absolute difference in in-hospital, 30-day and 1-year mortality rates between the two datasets differed less than 10% for 8 comorbidities and acute renal failure had an absolute difference of 16.0% for in-hospital mortality rate.

### Predicting Mortality in Administrative Data

The ability of the ICD-10 regression to discriminate between those deceased or alive was similar to the ICD-9 regression's ability, as shown by the C-statistics in Table 6. The C-statistics only varied between 0.81 and 0.83 for predicting in-hospital, 30-day, and 1-year mortality. Moreover, the odds ratios estimated by the ICD-9 and ICD-10 regressions showed similar model performance between the two datasets. The same result was observed when comparing the Brier scores between ICD-9 and ICD-10 regressions. The lower Brier scores for ICD-10 regressions relative to ICD-9 regression indicated that it performed slightly better than the ICD-9 regressions. Both ICD-9 and ICD-10 models explained approximately 20 percent of the variation in mortality, as shown by the R-squares in Table 6. The small difference between ICD-9 and ICD-10 models' R-squares shows again the similarity between the two coding systems.

All the regressions showed that patients who were 75 years or older were the most likely to die, relative to other age groups. The interpretation of the odds ratio for the gender variable differed across ICD-9 and ICD-10 regressions. For ICD-9 regressions, male patients were less likely to die than female patients, while the opposite result was found for ICD-10 regressions. Of the nine comorbidities, shock variable had the largest odds ratios in predicting in-hospital and 30-day mortality. The absolute difference in odds ratio between the two datasets was marginal for the comorbidities, differing by approximately 2%. All comorbidities were statistically significant at the 95% confidence level except for diabetes with complications in predicting 30-day mortality in ICD-10 data.

## Discussion

We developed the ICD-10 coding algorithms to define nine comorbidities used to adjust risk factors in AMI outcome studies. The newly developed ICD-10 coding algorithms were comparable with previously published and widely used ICD-9 coding algorithms in detection of cases and predicting mortality. We also know that the ICD-9 and 10 data had similar validity in recording the nine comorbidities when compared to chart review data.

The similar sensitivity, specificity, PPV and NPV found for the ICD-9 and ICD-10 coding algorithms when referenced to the chart review data suggests that both are similarly accurate in recording AMI comorbidities. However, both ICD-9 and ICD-10 datasets matched poorly to the chart review data for pulmonary edema, cardiac dysrhythmias, and cerebrovascular disease. The low sensitivities for pulmonary edema and cardiac dysrhythmias were likely due to the ambiguity of their diagnoses and/or being symptoms of underlying conditions [1,28-30]. In contrast to comorbidities that are well documented, such as cancer, physicians may document non-specific conditions or symptoms poorly in charts. Therefore, it is difficult for coders to make definitive decisions on the presence of these types of conditions based on the chart information, which can result in coding errors. The low positive predictive value for cerebrovascular disease is partly due to the low prevalence. Further, Benesch et al. [31] found a third of asymptomatic patients coded in ICD-9-CM as having cerebrovascular diseases, but found no evidence of occlusion or stenosis of the precerebral arteries [31]. Kokotailo and Hill [32] found that there was a range of coding errors for cerebrovascular disease, where transient ischemic attack (TIA) was coded for acute ischemic stroke. The difficulty with coding TIA was that the symptoms were non-specific, arising from many other conditions besides a temporary disruption in blood flow to a part of the brain [33]. Nevertheless, the similarity in misclassification by ICD-9 and ICD-10 coding algorithms for these three comorbidities suggests that risk-adjustment with the AMI nine comorbidities will not be affected by the change in coding system from ICD-9 to ICD-10.

When we applied the ICD-10 coding algorithms to the hospital discharge data, we found similarities across frequencies, mortality rates, and overall model performance to ICD-9 coding algorithms. This result suggests that the ICD-10 coding algorithms for these nine comorbidities performed similarly to past ICD-9 coding algorithms. This inference was further supported by the similar results found in our study and the study conducted by Tu et al. [3] Both studies found that the two most prevalent comorbidities were congestive heart failure and cardiac dysrhythmias, and the least prevalent comorbidity was pulmonary edema [3]. This similarity between the two studies was also shown by the overall model performances. Tu et al's 0.78 and 0.79 C-statistics for their 30-day and 1-year mortality logistic regressions, respectively matched closely to the 0.81 C-statistic we found in predicting for both mortality rates for our ICD-10 data [3]. Comparing our odds ratios to Tu et al.'s findings, we both found that patients with shock and acute renal failure tended to have the highest likelihoods of dying [3].

This study had some limitations. The first limitation was low frequencies for comorbidities in the chart review data, making it difficult to draw inferences to a broader population. Secondly, having chart review data from only teaching hospitals might generate a non-random sample, as it might have fewer healthy patients than a sample that included patients from non-teaching hospitals. Evidence of this result can be seen by the higher frequencies in the comorbidities in the Alberta chart review data relative to British Columbia discharge abstract data. Thirdly, gathering chart review data from the first year ICD-10 was implemented might result in a sample with high coding error. Fourthly, possible provincial and temporal changes in the occurrence of AMI, mortality, and comorbidities were not controlled. In the 10 year period, the criteria for AMI diagnosis were revised and efficient treatment to chronic disease was produced. For example, biomarkers of cardiac troponin and creatinine kinase-MB mass were included in diagnosis of myocardial infarction to increase sensitivity and specificity [34]. The advances in disease treatment and management can partly explain the slight variation between ICD-9 and ICD-10 models that can not be accounted for in our analysis. Fifthly, we excluded patients who were discharged on the day of admission or the following day. The limitation of this exclusion is that some true AMI cases were excluded. We re-analyzed our data after including deaths within 1 day. Including the 3,049 deaths within one day produced a decrease in the C-statistics for all our models. This suggests that the C-statistics reported in our study are slightly overestimated. Nevertheless, there remains a small difference between the predictive ability of ICD-9 and ICD-10 models. Lastly, the proportion of AMI patients that could not be linked with vital death registry or had moved out of the BC province without notice to the population registry during our study period were lost to follow-up. According to Li et al. (35), the accuracy of linkage between the registry file and inpatient discharge dataset was about 98%. We also suspect that only a few AMI patients moved out of the BC province given that our longest period of observing mortality was one year. Hence, our results were less likely affected by this small proportion of AMI patients lost to follow-up.

## Conclusion

The ICD-10 coding algorithms for AMI comorbidities produced in this study generated comparable results to past ICD-9 coding algorithms. This result was evident when ICD-9 and ICD-10 comorbidities were similar in frequencies and model performance. A step we took to ensure that not only did ICD-10 coding definitions matched well to past ICD-9 coding definitions, but also to a criterion standard, chart review data. The results from this step demonstrated comparable sensitivity, specificity, PPV and NPV between ICD-9 and ICD-10 coding algorithms. Evidence that the ICD-10 coding algorithms performed similar to past coding algorithms will allow future policy and quality judgements to be made on AMI outcomes with accurate ICD-10 comorbidities.

## Competing interests

The author(s) declare that they have no competing interests.

## Authors' contributions

LS contributed to the study design, statistical analysis, interpretation and writing of the manuscript. DE contributed to the study design, interpretation, and writing of the manuscript. HQ contributed to the study design, interpretation, and writing of the manuscript.

## Pre-publication history

The pre-publication history for this paper can be accessed here:



## Figures and Tables

**Table 1:** ICD-9 and ICD-10 Codes for AMI Comorbidities

**Comorbidities**	ICD-9*	**ICD-10**
Acute Myocardial Infarction	410.x	I21.x
Shock	785.5	R57
Diabetes with Complications	250.1–250.9	E10.0 – E10.8, E11.0 – E11.8, E12.0 – E12.8, E13.0 – E13.8, E14.0 – E14.8
Congestive Heart Failure	428.x	I09.9, I11.0, I13.0, I13.2, I25.5, I42.0, I42.5 – I42.9, I43.x, I50.x, P29.0
Cancer	140.0–208.9	C00.x–C97.x
Cerebrovascular disease	430.x–438.x	G45.x G46.x, H34.0, I60.x – I69.x
Pulmonary Edema	518.4, 514.x	J18.2, J81.x
Acute Renal Failure	584.x, 586.x, 788.5	N17.x, N19.x, R34.x
Chronic Renal Failure	585.x, 403.x, 404.x, 996.7, v451	N18.x, T82.4, Z49.2, Z99.2
Cardiac Dysrhythmias	427.x	I44.1–I44.3, I45.6, I45.9, I46.x, I47.x, I48.x, I49.x, R00.0, R00.1, R00.8, T82.1, Z45.0, Z95.0

*Note: ICD-9 codes are from Tu et al.[3]

**Table 2:** Prevalence of comorbidity in Alberta chart review data, ICD-9, and ICD-10 data among AMI patients (N = 193)

**Comorbidities**	**Chart Review**		**ICD-9 Data**		**ICD-10 Data**	
	N	% of 193	N	% of 193	N	% of 193
Shock	5	(2.6)	5	(2.6)	4	(2.1)
Diabetes with Complications	15	(7.8)	15	(7.8)	12	(6.2)
Congestive Heart Failure	55	(28.5)	50	(25.9)	47	(24.4)
Cancer	10	(5.2)	10	(5.2)	12	(6.2)
Cerebrovascular disease	6	(3.1)	6	(3.1)	6	(3.1)
Pulmonary Edema	28	(14.5)	2	(1.0)	2	(1.0)
Acute Renal Failure	15	(7.8)	15	(7.8)	20	(10.4)
Chronic Renal Failure	18	(9.3)	22	(11.4)	16	(8.3)
Cardiac Dysrhythmias	106	(54.9)	33	(17.1)	40	(20.7)

**Table 3:** Agreement between ICD-9/ICD-10 and Alberta chart review data among AMI patients (N = 193)

**Comorbidities**	**ICD-9 Data**				**ICD-10 Data**			
	Sensitivity	Specificity	PPV	NPV	Sensitivity	Specificity	PPV	NPV
Shock	60.0	98.9	60.0	98.9	60.0	99.5	75.0	98.9
(95% CI)	(14.66 – 94.73)	(96.21 – 99.87)	(14.66 – 94.73)	(96.21 – 99.87)	(14.66 – 94.73)	(97.07 – 99.99)	(19.41 – 99.37)	(96.23 – 99.87)
Diabetes with Complications	80.0	98.3	80.0	98.3	66.7	98.9	83.3	97.2
(95% CI)	(51.91 – 95.67)	(95.15 – 99.65)	(51.91 – 95.67)	(95.15 – 99.65)	(38.38 – 88.18)	(96.00 – 99.86)	(51.59 – 97.91)	(93.67 – 99.10)
Congestive Heart Failure	81.8	96.4	90.0	93.0	80.0	97.8	93.6	92.5
(95% CI)	(69.10 – 0.92)	(91.75 – 98.81)	(78.19 – 96.67)	(87.52 – 96.60)	(67.03 – 89.57)	(93.78 – 99.55)	(82.46 – 98.66)	(86.92 – 96.18)
Cancer	90.0	99.5	90.0	99.5	90.0	98.4	75.0	99.4
(95% CI)	(55.50 – 99.75)	(96.99 – 99.99)	(55.50 – 99.75)	(96.99 – 99.99)	(55.50 – 99.75)	(95.28 – 99.66)	(42.81 – 94.51)	(96.96 – 99.99)
Cerebrovascular disease	100.0	93.6	33.3	100.0	100.0	95.7	42.9	100.0
(95% CI)	(54.07 – 100.00)	(89.06 – 96.64)	(13.34 – 59.01)	(97.91 – 100.00)	(54.07 – 100.00)	(91.74 – 98.14)	(17.66 – 71.14)	(97.96 – 100.00)
Pulmonary Edema	7.1	100.0	100.0	86.4	7.1	100.0	100.0	86.4
(95% CI)	(0.88 – 23.50)	(97.79 – 100.00)	(15.81 – 100.00)	(80.69 – 90.91)	(0.88 – 23.50)	(97.79 – 100.00)	(15.81 – 100.00)	(80.69 – 90.91)
Acute Renal Failure	80.0	98.3	80.0	98.3	80.0	95.5	60.0	98.3
(95% CI)	(51.91 – 95.67)	(95.15 – 99.65)	(51.91 – 95.67)	(95.15 – 99.65)	(51.91 – 95.67)	(91.34 – 98.04)	(36.05 – 80.88)	(95.02 – 99.64)
Chronic Renal Failure	83.3	96.0	68.2	98.2	72.2	98.3	81.3	97.2
(95% CI)	(58.58 – 96.42)	(91.93 – 98.38)	(45.13 – 86.14)	(94.96 – 99.64)	(46.52 – 90.31)	(95.07 – 99.65)	(54.35 – 95.95)	(93.53 – 99.08)
Cardiac Dysrhythmias	35.9	100.0	100.0	56.1	35.8	97.7	95.0	55.6
(95% CI)	(26.77 – 45.74)	(95.85 – 100.00)	(90.75 – 100.00)	(47.94 – 64.08)	(26.77 – 45.74)	(91.94 – 99.72)	(83.08 – 99.39)	(47.31 – 63.58)

**Table 4:** Prevalence of comorbidities among AMI patients (%)

**Variables**	**ICD-9 Data**		**ICD-10 Data**	
	N	(% of 29610)	N	(% of 20251)
**Age**				
20 – 49	2835	(9.6)	1895	(9.4)
50 – 64	8136	(27.5)	5499	(27.2)
65 – 74	8151	(27.5)	4793	(23.7)
>=75	10488	(35.4)	8064	(39.8)
				
**Gender**				
Female	9812	(33.1)	6976	(34.4)
Male	19798	(66.9)	13275	(65.6)
				
**Comorbidities**				
Shock	749	(2.5)	366	(1.8)
Diabetes with Complications	510	(1.7)	535	(2.6)
Congestive Heart Failure	6319	(21.3)	3963	(19.6)
Cancer	512	(1.7)	362	(1.8)
Cerebrovascular Disease	1027	(3.5)	510	(2.5)
Pulmonary Edema	188	(0.6)	119	(0.6)
Acute Renal Failure	632	(2.1)	720	(3.6)
Chronic Renal Failure	783	(2.6)	695	(3.4)
Cardiac Dysrhythmias	5540	(18.7)	3478	(17.2)

**Table 5:** Mortality by comorbidities among AMI patients from the BC provincial database (N = 49,861)

Variables	**ICD-9 Data** In-hospital		**ICD-10 Data** In-hospital		**ICD-9 Data** 30-day		**ICD-10 Data** 30-day		**ICD-9 Data** 1 Year		**ICD-10 Data** 1 Year	
	N	% of 29610	N	% of 20251	N	% of 29610	N	% of 20251	N	% of 29610	N	% of 20251
**Overall**	2384	8.1	1386	6.8	2943	9.9	1824	9.0	4899	16.5	3311	16.3
												
**Age**												
20 – 49	26	0.9	23	1.2	38	1.3	29	1.5	64	2.3	46	2.4
50 – 64	194	2.4	100	1.8	260	3.2	152	2.8	449	5.5	271	4.9
65 – 74	501	6.1	203	4.2	659	8.1	292	6.1	1108	13.6	548	11.4
>=75	1663	15.9	1060	13.1	1986	18.9	1351	16.8	3278	31.3	2446	30.3
												
**Gender**												
Female	1136	11.6	580	8.3	1343	13.7	754	10.8	2139	21.8	1401	20.1
Male	1248	6.3	806	6.1	1600	8.1	1070	8.1	2760	13.9	1910	14.4
												
**Comorbidities**												
Shock	394	52.6	173	47.3	407	54.3	178	48.6	438	58.5	194	53.0
Diabetes with Complications	80	15.7	71	13.3	94	18.4	83	15.5	167	32.7	166	31.0
Congestive Heart Failure	1205	19.1	665	16.8	1435	22.7	856	21.6	2378	37.6	1501	37.9
Cancer	91	17.8	70	19.3	109	21.3	103	28.5	230	44.9	202	55.8
Cerebrovascular disease	267	26.0	130	25.5	293	28.5	149	29.2	424	41.3	209	41.0
Pulmonary Edema	50	26.6	25	21.0	56	29.8	33	27.7	81	43.1	53	44.5
Acute Renal Failure	298	47.2	224	31.1	307	48.6	253	35.1	380	60.1	358	49.7
Chronic Renal Failure	167	21.3	122	17.6	184	23.5	167	24.0	310	39.6	308	44.3
Cardiac Dysrhythmias	940	17.0	506	14.5	1057	19.1	596	17.1	1588	28.7	972	27.9

Note: Both ICD-9 and ICD-10 data excluded all patients who were discharged on the same day of admission or the following day

**Table 6:** Risk adjusted odds ratios (OR) for mortality in ICD-9 and ICD-10 data among AMI patients

	**ICD-9 Data** In-hospital		**ICD-10 Data** In-hospital		**ICD-9 Data** 30-Day		**ICD-10 Data** 30-Day		**ICD-9 Data** 1 Year		**ICD-10 Data** 1 Year	
Variables	OR	(95% CI)	OR	(95% CI)	OR	(95% CI)	OR	(95% CI)	OR	(95% CI)	OR	(95% CI)
**Age**												
20 – 49	1.0	(1.0–1.0)	1.0	(1.0–1.0)	1.0	(1.0–1.0)	1.0	(1.0–1.0)	1.0	(1.0–1.0)	1.0	(1.0–1.0)
50 – 64	2.1	(1.4–3.2)	1.1	(0.7–1.8)	2.0	(1.5–2.8)	1.5	(1.0–2.2)	2.1	(1.6–2.8)	1.7	(1.3–2.4)
65 – 74	4.4	(2.9–6.7)	2.3	(1.5–3.6)	4.3	(3.1–6.1)	2.8	(1.9–4.2)	4.6	(3.5–5.9)	3.6	(2.7–5.0)
>=75	11.4	(7.6–17.14)	7.5	(4.9–11.6)	10.1	(7.2–14.1)	8.0	(5.5–11.7)	11.6	(9.0–15.0)	11.0	(8.1–14.9)
												
**Gender**												
Female	1.0	(1.0–1.0)	1.0	(1.0–1.0)	1.0	(1.0–1.0)	1.0	(1.0–1.0)	1.0	(1.0–1.0)	1.0	(1.0–1.0)
Male	0.7	(0.7–0.8)	1.0	(0.9–1.2)	0.8	(0.8–0.9)	1.1	(1.0–1.2)	0.9	(0.8–1.0)	1.0	(0.9–1.1)
												
**Comorbidities**												
Shock	10.0	(8.4–11.9)	10.4	(8.1–13.4)	8.1	(6.9–9.6)	7.6	(6.0–9.7)	4.8	(4.1–5.7)	4.2	(3.3–5.4)
Diabetes with Complications	1.5	(1.1–2.0)	1.4	(1.0–1.9)	1.5	(1.1–1.9)	1.2	(0.9–1.5)	2.0	(1.6–2.5)	1.5	(1.2–1.9)
Congestive Heart Failure	2.1	(1.9–2.3)	2.2	(1.9–2.4)	2.1	(2.0–2.3)	2.3	(2.1–2.6)	2.7	(2.5–2.9)	2.8	(2.5–3.0)
Cancer	2.2	(1.7–2.8)	2.4	(1.8–3.2)	2.1	(1.7–2.7)	3.1	(2.4–4.1)	3.9	(3.2–4.8)	5.8	(4.6–7.4)
Cerebrovascular disease	3.0	(2.6–3.6)	3.5	(2.8–4.5)	2.6	(2.3–3.1)	3.1	(2.5–3.9)	2.6	(2.2–3.0)	2.6	(2.1–3.2)
Pulmonary Edema	2.6	(1.8–3.8)	2.5	(1.5–4.1)	2.5	(1.7–3.6)	2.9	(1.8–4.5)	2.8	(2.0–3.9)	3.4	(2.2–5.1)
Acute Renal Failure	5.1	(4.2–6.2)	3.1	(2.6–3.8)	4.2	(3.5–5.0)	2.7	(2.3–3.3)	3.5	(2.9–4.3)	2.4	(2.0–2.8)
Chronic Renal Failure	1.9	(1.6–2.4)	1.4	(1.1–1.8)	1.7	(1.4–2.1)	1.7	(1.4–2.1)	2.1	(1.8–2.5)	2.2	(1.8–2.6)
Cardiac Dysrhythmias	2.0	(1.9–2.3)	1.8	(1.6–2.1)	1.8	(1.6–1.9)	1.6	(1.4–1.8)	1.6	(1.5–1.8)	1.5	(1.3–1.6)
Constant	0.0	(0.0–0.0)	0.0	(0.0–0.0)	0.0	(0.0–0.0)	0.0	(0.0–0.0)	0.0	(0.0–0.0)	0.0	(0.0–0.0)
												
C-Statistic (AUC)	0.841		0.826		0.816		0.809		0.815		0.812	
Brier Score	0.062		0.055		0.062		0.056		0.074		0.072	
R-square	0.224		0.201		0.195		0.182		0.207		0.207	
												
Number of patients	29610		20251		29610		20251		29610		20251	

Note: All Odds Ratios (OR) adjust for age, sex, shock, diabetes with complications, congestive heart failure, cancer, cerebrovascular disease, pulmonary edema, acute renal failure, chronic renal failure, and cardiac dysrhythmias.
